# Higher-than-expected prevalence of non-tuberculous mycobacteria in HIV setting in Botswana: Implications for diagnostic algorithms using Xpert MTB/RIF assay

**DOI:** 10.1371/journal.pone.0189981

**Published:** 2017-12-22

**Authors:** Tefera Agizew, Joyce Basotli, Heather Alexander, Rosanna Boyd, Gaoraelwe Letsibogo, Andrew Auld, Sambayawo Nyirenda, Zegabriel Tedla, Anikie Mathoma, Unami Mathebula, Sherri Pals, Anand Date, Alyssa Finlay

**Affiliations:** 1 Centers for Disease Control and Prevention, Gaborone, Botswana; 2 Centers for Disease Control and Prevention, Division of Global HIV and TB, Atlanta, Georgia, United States of America; 3 Centers for Disease Control and Prevention, Division of Tuberculosis Elimination, Atlanta, Georgia, United States of America; 4 National Tuberculosis Reference Laboratory, Ministry of Health, Gaborone, Botswana; Universidad Nacional de la Plata, ARGENTINA

## Abstract

**Background:**

Non-tuberculous mycobacteria (NTM) can cause pulmonary infection and disease especially among people living with HIV (PLHIV). PLHIV with NTM disease may clinically present with one of the four symptoms consistent with tuberculosis (TB). We describe the prevalence of NTM and *Mycobacterium tuberculosis complex* (MTBC) isolated among PLHIV who presented for HIV care and treatment.

**Methods:**

All PLHIV patients presenting for HIV care and treatment services at 22 clinical sites in Botswana were offered screening for TB and were recruited. Patients who had ≥1 TB symptom were asked to submit sputa for Xpert MTB/RIF and culture. Culture growth was identified as NTM and MTBC using the SD-Bioline TB Ag MPT64 Kit and Ziehl Neelsen microscopy. NTM and MTBC isolates underwent species identification by the Hain GenoType CM and AS line probe assays.

**Results:**

Among 16, 259 PLHIV enrolled 3068 screened positive for at least one TB symptom. Of these, 1940 submitted ≥1 sputum specimen, 427 (22%) patients had ≥1 positive-culture result identified phenotypically for mycobacterial growth. Of these 247 and 180 patients were identified as having isolates were NTM and MTBC, respectively. Of the 247 patients identified with isolates containing NTM; 19 were later excluded as not having NTM based on additional genotypic testing. Among the remaining 408 patients 228 (56%, 95% confidence interval, 46–66%) with NTM. *M*. *intracellulare* was the most common isolated (47.8%). Other NTMs commonly associated with pulmonary disease included *M*. *malmoense* (3.9%), *M*. *avium* (2.2%), *M*. *abscessus* (0.9%) and *M*. *kansasii* (0.4%). After excluding NTM isolates that were non-speciated and *M*. *gordonae* 154 (67.5%) of the NTM isolates were potential pathogens.

**Conclusions:**

In the setting of HIV care and treatment, over-half (56%) of a positive sputum culture among PLHIV with TB symptoms was NTM. Though we were not able to distinguish in our study NTM disease and colonization, the study suggests culture and species identification for PLHIV presenting with TB symptoms remains important to facilitate NTM diagnosis and hasten time to appropriate treatment.

## Introduction

Non-tuberculous mycobacteria (NTM) can cause pulmonary infection and disease especially among people living with HIV (PLHIV). PLHIV with NTM infection may clinically present with one of the four symptoms (cough, fever, night sweat and weight loss) consistent with tuberculosis (TB).

Emerging evidences suggests that rate of NTM case reports among PLHIV is increasing in Africa and other parts of the world [1, 2]. Reports from a few sub-Saharan laboratories indicate that the prevalence of NTM among samples with a culture-positive for mycobacteria ranges from 4–39%. The African studies described sputum samples collected from both HIV infected and uninfected populations [3–7] with presumptive TB. There are limited published reports on the type of species and the geographic distribution of NTM isolated from pulmonary samples among patients with presumptive TB globally [8] and in particular, data are scarce in Africa [9].

Hoza *et*.*al* (Tanzania) and Aliyu *et*.*al* (Nigeria) reported that *M*. *intracellulare and M*. *gordonae* were among the top three species identified [1, 6]. While factors such as an increase in the aging population and prevalent chronic diseases could contribute to the increase in the prevalence of NTM in industrialized countries, HIV infection and the associated immune-compromised state is a likely contributor in many African countries [2].

Due to laboratory capacity challenges, data are limited on the prevalence of NTM infection or disease in low and middle income countries [6]. In recent years the availability of the Hain GenoType CM and AS line probe assays (LPA) facilitated NTM species identification [10] in resource-limited settings. Since pulmonary disease caused by NTM and *Mycobacterium tuberculosis complex (*MTBC) can have similar clinical presentations, NTM characterization is essential to minimize misclassification, misdiagnosis and thus delivery of appropriate clinical management to patients with NTM and MTBC infection or disease.

The Xpert MTB/RIF assay has high sensitivity for detecting pulmonary MTBC and has very high specificity, >99%, to rule out TB. The specificity of Xpert MTB/RIF among symptomatic NTM-positive patients, however, ranges from 92% to 99% [3, 11, 12] with possibility of higher Xpert MTB/RIF false positivity. Helb *et al* in 2010 also reported that false Xpert MTB/RIF rifampicin resistance with one NTM species (*M*. *malmoense*) was a possibility because of weak cross hybridization [13]. The current TB diagnostic algorithms in Botswana do not account for a possible NTM diagnosis and management [14] among symptomatic patients with a negative Xpert MTB/RIF test result.

Our study aims to describe: (1) the prevalence of NTM and MTBC from sputum samples collected from PLHIV with presumptive TB in Botswana, (2) the types of NTMs identified, and (3) the specificity of Xpert MTB/RIF among isolates from samples culture-positive for NTM.

## Methods

This is a sub-study of the Xpert Package Rollout Evaluation Study using a Stepped-wedge design (XPRES) [15]. Full details of the study protocol, including study populations, sample size, and procedures can be accessed in the published protocol.

All HIV infected patients presenting for HIV care and treatment services at 22 clinical sites in Botswana were offered screening for TB and were recruited from August 2012 through November 2014. For patients who screened positive for one or more any duration of TB symptoms (cough, fever, night sweats and weight loss) demographic data [CD4 count, body mass index (BMI) and history of TB] were collected and patients were asked to submit four sputum samples: 2 spots specimens (1 and 2) on the day of screening and 1 morning and 1 spot (spot 3) on the following day.

Spot specimens one and three were tested by Xpert MTB/RIF and Ziehl Neelsen (ZN) smear microscopy at the peripheral laboratory. Morning and spot two specimens were transported to the National TB Reference Laboratory (NTRL) for culture and drug susceptibility testing (DST). Similar methods reported by Aliyu *et al* [6] were used to process and test at NTRL. Sputum samples were treated with BD Mycoprep (Beckton Dickinson, Sparks, Maryland, United States of America (USA)) which consists of 1% N-acetyl-L-cysteine (NALC), 4% sodium hydroxide and 2.9% sodium citrate then incubated in the automated BACTEC MGIT 960 instrument (Becton Dickinson, Sparks, Maryland, USA).

Samples that failed to show any growth after 42 days of incubation in the MGIT 960 were removed and classified as negative based on the manufacturer protocol. Samples with positive growth were removed from the instrument and inoculated on blood agar to check for non-mycobacterial contamination. Then, a ZN smear was performed to check for the presence of Acid Fast Bacilli (AFB).

Cultures with positive growth in the MGIT 960 and presence of AFB by ZN were tested with a rapid TB immunochromatographic assay (SD-Bioline Ag MPT64 RapidTM assay, Standard Diagnostics, Kyonggi-do, Korea) to discriminate between NTM and MTBC.

Cultures with positive growth in the MGIT 960 and presence of AFB but that were negative for MTBC using the SD-Bioline assay were sub-cultured on Lowenstein Jensen (LJ) media. Those that subsequently grew on LJ medium were considered to be presumptive NTMs, and were characterized to species level with LPA (GenoType CM and AS assays, Hain Lifescience, Nehren, Germany) according to manufacturer recommendations.

To describe the specificity of Xpert MTB/RIF in our setting, all patient who had a culture-positive isolate identified as NTM were tested with Xpert MTB/RIF retrospectively. To describe the geographic distribution of we recorded NTN positive cultures by origin of samples in the 12 districts included in the study.

During the study, an investigation of potential sources of environmental NTM at the NTRL was conducted to rule out contamination from water sources. NTRL is an accredited laboratory where both internal and external quality assurance procedures are regularly conducted. Laboratory water sources were sampled for five consecutive days and processed as per standard methods [16, 17]. Both tap water in the media preparation room and water from the deionizer that was used to prepare all reagents were included. If a culture result turned NTM positive (phenotypically) from a NTRL water source, further testing was conducted using LPA (molecular) to identify NTM species.

### Data analysis

We used STATA (version 14.0, Stata Corp, Collage Station, TX, USA) [18] to fit univariate and multiple logistic regression models to compare demographic and clinical characteristics between patients with MTBC and NTM, adjusted for within-clinic correlation. *P* value of <0.05 was considered statistically significant. We also used descriptive comparison of geographic mapping of origin of NTMs among the 12 districts included in the study.

### Ethical considerations

The study protocol was approved by the Botswana Health Research and Development Committee (May 16, 2012), the USA Centers for Disease Control and Prevention Institutional Review Board (IRB) (July 19, 2012), and the University of Pennsylvania IRB (June 24, 2012). Patients at study sites were enrolled in the study following the IRB-approved, written, informed consent process.

## Results

**Fig 1:**
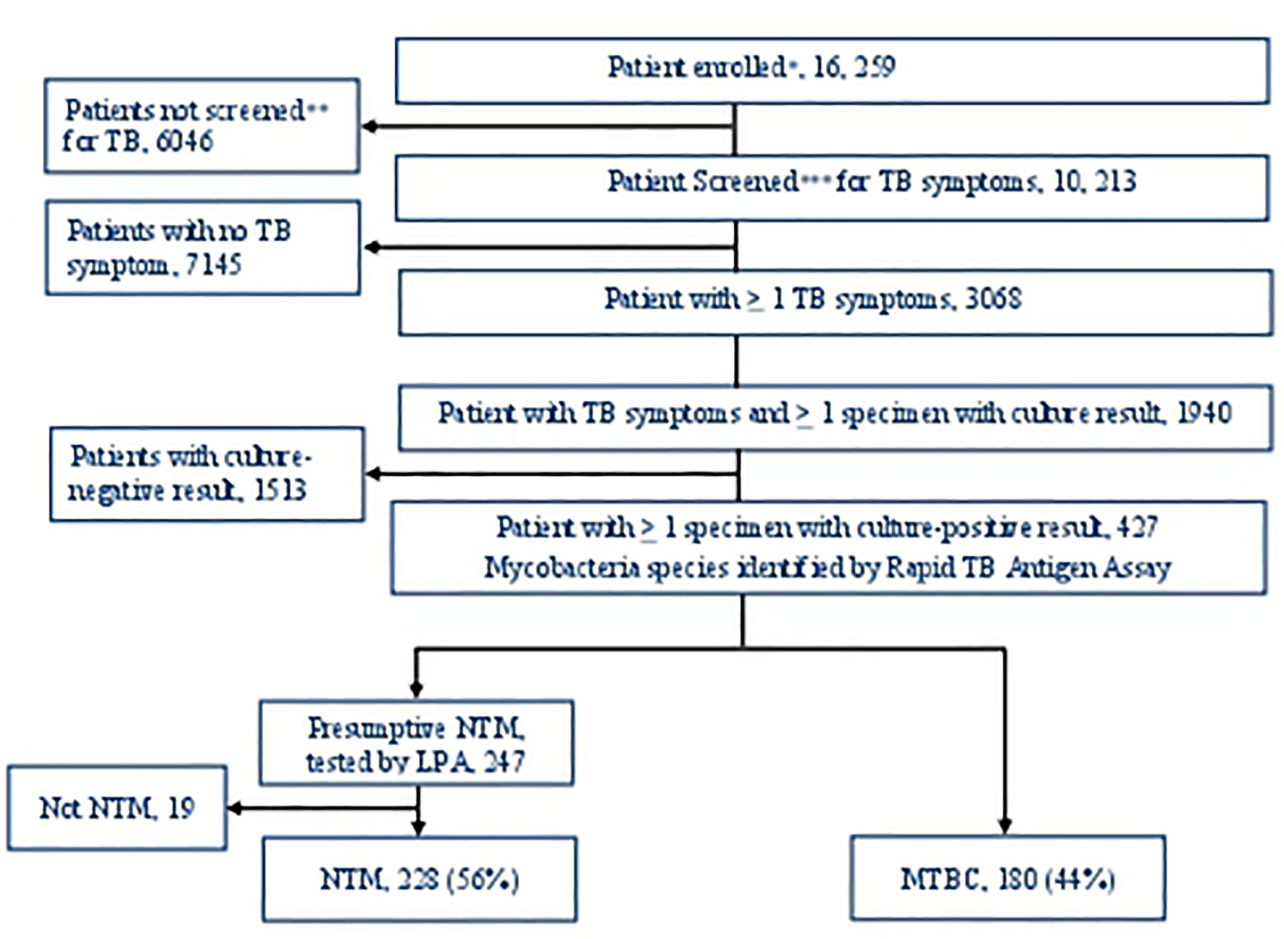
“Patients with at least one sputum culture results by mycobacterium species August 2012 –November 2014” Notes: * Of the total 16, 259, the 6041 were enrolled prospectively and the 10, 218 retrospectively; ** only 10, 213 were screened for TB symptoms due to amendment on main study (XPRES) data collection procedure; *** of the 3068 screened positive for TB symptom, 2296 were among prospective and 772 from retrospective cohort.

From August 2012 to November 2014, 16, 259 PLHIV enrolled and 10, 213 were screened for TB symptoms and for the remaining 6046 only data abstraction was done due to amendment to main study (XPRES) data collection procedure. Among patients screened for TB symptoms 3068 screened positive for ≥ 1 TB symptom. Of these, 1940 patients were able to submit sputum specimens for both culture and Xpert MTB/RIF testing. The median age was 37 (interquartile range, IQR 31–45), 1147/1940 (59%) were female, and the median baseline CD4 count was 249 cells/mm^3^ (IQR 127–340). Among 1940, 427 (22%) had ≥1 specimen with a positive-culture result by MGIT 960. Of these 247 and 180 were identified phenotypically as having isolates with NTM and MTBC, respectively. Nineteen of the 247 patients initially identified as NTM were later excluded as NTM based on additional genotypic testing with LPA. One of the patients with NTM was also identified with additional MTBC after genotypic testing. Of the remaining 408 patients with identified species 228 (56%, 95% confidence interval (CI), 46–66%) were NTM and 180 (44%, 95% CI, 39–49%) were MTBC (As shown in Fig 1). The proportion of patients who had two culture positives for NTM and MTBC, respectively, were 3.5% (8/228) vs. 16.7% (30/180), OR = 0.18, 95% CI, 0.08–0.40, *p<0*.*001*.

Demographic characteristics, CD4 count, BMI, history of TB and presenting clinical features were similar among patients with culture-positive NTM and MTBC, except age ≥50 that was higher among patients with culture-positive NTM (19.7% vs. 12.2%, aOR, 1.67, *p <0*.*044*). Anemia (Haemoglobin, Hgb, <10mg/dl) was less common among patients with culture-positive NTM culture than culture-positive MTBC (15.7% vs. 31.4%, aOR, 0.26, *p = 0*.*015)*. Patients with culture-positive NTM were significantly less likely to have a positive smears than culture-positive MTBC patients (6.1% vs. 54.4%, aOR, 0.04, *p <0*.*001*) (Table 1). Proportion of patients receiving Anti-retroviral therapy among NTM and MTBC group, respectively, was 67 (29.4%) and 39 (21.7%), unOR 1.50, *p = 0*.*078*.

**Table 1:** Characteristics of patients with positive-culture for NTM and MTBC in a multilevel logistic regression model.

** **			** **								
	NTM†			MTBC‡							
Characteristics	N	N	(%)	N	n	(%)	Crude OR	P value	aOR§	(95% CI≠)	P value
Age* ≥50	228	45	19.7%	180	22	12.2%	1.77	0.020	1.67	1.02–2.74	0.044
Gender, female	228	123	53.9%	180	86	47.8%	1.28	0.255	1.10	0.56–2.34	0.657
CD4** count <200	220	97	44.1%	177	93	52.5%	0.71	0.109	1.57	0.82–3.00	0.147
BMI <18.5	214	70	32.7%	180	71	39.4%	0.75	0.346	1.30	0.64–2.62	0.438
Hgb <10 mg/dl	191	30	15.7%	156	49	31.4%	0.41	0.024	0.26	0.09–0.73	0.016
TB Symptoms											
Cough	220	152	69.1%	180	145	80.6%	0.54	0.066	0.71	0.23–2.17	0.527
Fever	219	52	23.7%	180	87	48.3%	0.33	0.003	0.48	0.17–1.35	0.152
Night sweats	220	57	25.9%	180	84	46.7%	0.40	0.007	0.94	0.30–2.97	0.916
Weight loss	221	93	42.1%	180	123	68.3%	0.34	0.005	0.64	0.26–1.58	0.313
History of TB, Yes	218	27	12.4%	180	23	12.8%	0.96	0.896	1.60	0.47–5.49	0.440
Smear microscopy	228	14	6.1%	180	98	54.4%	0.05	<0.001	0.04	0.02–0.10	<0.001
positive											

* Median age among patients with NTM and MTBC = 37 years.

** Median CD4 among patients with NTM and MTBC were 233 and 190, respectively. And CD4 <50 among patients with NTM and MTBC, respectively, with available data were 14.5% (32/220) and 11.9% (21/177), unOR = 1.26, *P = 0*.*435*.

† Non-tuberculous mycobacteria = NTM

‡ Mycobacterium tuberculous complex = MTBC

§ Adjusted odds ratio = aOR

≠ Confidence interval = CI

### Species identification and Xpert MTB/RIF result for NTM isolates

NTM species were identified in sputum samples from 180/228 (78.9%) patients, including 7 mixed NTM species. *M*. *intracellulare* was the most common species isolated 109 (47.8%). Other NTMs commonly associated with pulmonary disease included *M*. *malmoense* 9 (3.9%), *M*. *avium* 5 (2.2%), *M*. *abscessus* 2 (0.9%), and *M*. *kansasii* 1 (0.4%). The common environmental contaminant *M*. *gordonae* was identified in 16 patients (7%). Forty-eight (21.1%) NTMs could not be speciated by the current Hain GenoType CM and AS LPA that we used for testing. After excluding NTM isolates that were non-speciated and *M*. *gordonae*, 154 (67.5%) of the NTM isolates were potential pathogens. (Table 2). Over the study period no temporal trends were noted in terms of isolation of NTM (S1 Fig).

**Table 2:** NTM isolated among PLHIV presenting with TB symptoms in Botswana and corresponding Xpert MTB/RIF result.

NTM Species	Number	Frequency	Xpert MTB/RIF Results			
			MTB detected	MTB not detected	Not available	RIF resistant
*M*. *intracellulare*	109	47.8%	0	109	0	0
*M*. *gordonae*	16	7.0%	0	16	0	0
*M*. *malmoense*	9	3.9%	0	9	0	0
*M*. *simiae*	8	3.5%	0	8	0	0
*M*. *scrofulaceum*	6	2.6%	0	6	0	0
*M*. *fortuitum*	6	2.6%	0	6	0	0
*M*. *asiaticum*	6	2.6%	0	6	0	0
*M*. *avium*	5	2.2%	0	5	0	0
*M*. *genavense*	2	0.9%	0	2	0	0
*M*. *lentiflavum*	2	0.9%	0	2	0	0
*M*. *abscessus*	2	0.9%	0	2	0	0
*M*. *kansasii*	1	0.4%	0	1	0	0
*M*. *phlei*	1	0.4%	0	1	0	0
Mixed NTM*	7	3.1%	1	6	0	1
Others^†^	48	21.1%	2	37	9	2
**Total**	**228**	**100.00%**	3	216	9	3

*Mixed species: more than one NTM species identified per isolate

^†^NTMs ‘that we were not able to speciate further using the current testing methods we used’

Among the 408 isolates from culture-positive specimens tested retrospectively, Xpert MTB/RIF test results were available for 219/228 (96.1%) of NTM isolates and 177/180 (98.3%) of MTBC isolates. For three patients with NTM species (1.4%) the Xpert MTB/RIF result indicated MTB detected with rifampicin resistance. One of these three patients with NTM species from which MTBC was detected had a mixed NTM (*M*. *avium and M*. *intracellulare*). The other two had mycobacterium species that we were not able to speciate further with the current testing methods we used. The specificity of Xpert MTB/RIF in this setting of PLHIV with NTM isolate was 216/219 (98.6%) (Table 2). The sensitivity of Xpert MTB/RIF among PLHIV with culture and genotypically confirmed MTBC isolates was 176/177 (99.4%).

### Geographic distribution of NTM by district

**Fig 2:**
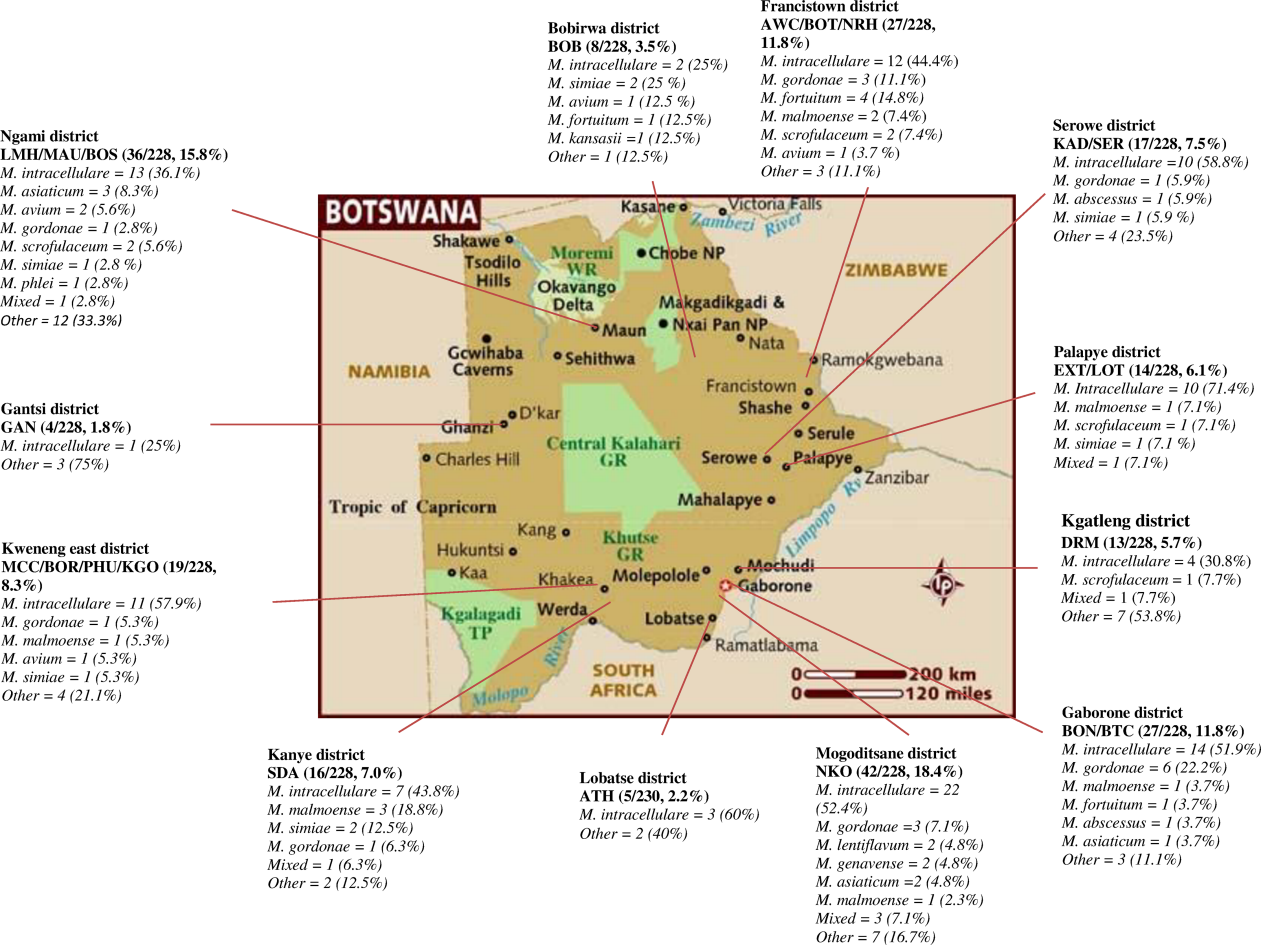
“Distribution of NTM among symptomatic PLHIV by district and clinical sites". **Key to Fig 2:** Athlon Hospital = ATH, Area W Clinic = AWC, Bontleng Clinic = BON, Borakalalo Clinic = BOR, Boseja Clinic = BOS, Botswelelo Clinic = BOT, Broadhurst Traditional Clinic = BTC, Bobonong Primary Hospital = BOB, Deborah Retief Memorial Hospital = DRM, Ext 3 Clinic = EXT, Gantsi Hospital = GAN, Kadimo Clinic = KAD, Letsholathebe II Memorial Hospital = LMH, Lotsane Clinic = LOT, Maun Clinic = MAU, Molepolole Council Clinic = MCC, Kgosing Clinic = KGO, Nkoyaphiri Clinic = NKO, Nyangabgwe Referral Hospital = NRH, Phuthdikobo Clinic = PHU, SDA Hospital = SDA and Serowe Clinic = SER.

Fig 2 displays the geographic distribution of NTM species. Twelve out of the 28 districts were included in this study. *M*. *intracellulare* the most isolated NTM was identified in all the 12 districts, while *M*. *gordonae and M*. *malmoense*, the second and third most common were found in 7/12 (58.3%) and 6/12 (50%) of the districts, respectively. *M*. *simiae* was found in *4/12 (33*.*3%)*, *M*. *avium and M*. *scrofulaceum* were in 3/12 (25%), *M*. *kansasii*, *M*. *phlei*, *M*. *genavense and M*. *lentiflavum* were in only one out of the 12 districts.

A total of 40 water samples were cultured from the water source used in the media preparation room and also water from the deionizer that was used to prepare reagents. Ten percent (4/40) of results phenotypically indicated NTM positive-culture. When further tested using Hain GenoType CM/AS line probe assays no NTM was isolated ruling out contamination from the source of water used for the preparation of all reagents and processing in the culture laboratory.

## Discussion

The results of this study demonstrated that among culture-positive sputum specimens collected from PLHIV with TB symptoms, 56% grew NTM. To our knowledge, this prevalence of NTM among culture-positive specimens from PLHIV with TB symptoms is the highest described to date [5, 19, 20, 23], particularly in high HIV prevalence settings. Previous publications from African countries reported NTM prevalence among samples with a positive culture for mycobacteria ranged from 4–39% [3–7]. Unlike our study, the TB screening criteria used for most was ≥ 2 weeks of TB symptoms and the populations studied were mixed, including both HIV-infected and un-infected persons. The four symptom TB screening was used by Aliyu et al and the NTM prevalence was 16% among HIV infected and uninfected population of in-patient and out-patient.

In recent years, a few large population based TB prevalence surveys both in Africa and Asia, have reported a high proportion of NTM (45–89%) among presumptive TB patients with sputum positive for mycobacteria by MGIT 960 [21, 22]. In these settings participants with presumptive TB were defined both by presence of symptoms and/or chest radiography with an abnormality in the lung or pleura. These surveys also included both persons with and without HIV infection. Given the different population and screening criteria of the above studies, these results are not directly comparable to our study.

Sonnenberg, Conde and Lan *et als* reported 8%, 9.7% and 47% NTM prevalence among HIV infected patients with positive mycobacterial cultures, from South Africa, Brazil and China, respectively [5, 19, 20]. The sputum specimens in the China study, that has closer prevalence with the present study, were collected from patients with CD4 counts < 350 cells/mm^3^, and had slightly different methods: the TB symptom screening methods used were before the current four symptom TB screen was recommended by WHO and the culture methods used was on a BacT/Alert 3D Microbial Detection System (bioMerieux, Craponne, France). In contrast to the report from Lan *et al* [19] our patients had higher median CD4 lymphocyte counts (205 vs. 23 cells/mm^3^). Given the higher risk of NTM with advanced HIV disease in Lan *et al* report and the higher median CD4 lymphocyte count in our study the prevalence of NTM is higher than expected. Compared to the present study Sonnenberg *et al* and Conde *et al* reported lower NTM proportion from the HIV infected sub-group of studied population. The Sonnenberg *et al* report was a case-control study amongst gold miner with a positive sputum mycobacterial culture tested using earlier version of liquid medium (Broth culture systems such as the BACTEC 460 (Becton Dickinson Diagnostic Instruments, Sparks, Md.). While Conde *et al* reported from hospitalized patients, used solid culture medium (Lowenstein-Jensen) and the HIV testing was not offered to all patients rather to those with risk factors for AIDS with symptoms. Moreover, similar to China report both in Sonnenberg *et al* and Conde *et al* the TB symptom screening method used was not four symptom TB screen. In the present analysis direct comparison with these previous studies was not done because of the various studied population, TB screening and culture methods used.

Liquid culture has been scaled up globally and increasingly used as the reference standard for confirming MTBC by referral laboratories. Use of liquid based culture methods are known to recover more NTM than solid based culture methods among persons with presumptive TB, including in high HIV prevalence settings [24] and this may contribute to the increased identification and reporting of NTM from large studies.

Botswana has the second highest HIV prevalence in the world: 19% in the general population [25]. Given the potential concern of pulmonary NTM infection and disease and unknown burden in this population, the findings have potential program implications. In view of high HIV prevalence in the present study setting and rising NTM infection and disease in Africa the findings are important to the larger global community [1, 7, 26]. HIV infection is a key risk factor [1, 19] that might explain of such high NTM prevalence in our study. Other potential reason such as prevalent chronic diseases was not included in the analysis since it was beyond the scope of the present study. Yet another area considered was contamination and an investigation of potential laboratory water sources of contamination at our referral laboratory indicated contamination during processing as less likely [27]. L. Buijtels *et al* from Zambia reported from a controlled study that the estimated rate of colonization in a mixed population of HIV infected and uninfected patients was 9% and the rate of disease was ≈2% [7]. While we acknowledge the potential clinical relevance of NTM colonization, infection and disease, since the present study was not designed to address NTN diagnosis the America Thoracic Society criteria was not used and thus distinguishing NTM colonization versus disease was not possible. However, in view of the epidemiology of NTM in Southern Africa, the high TB case notification rates (over 408/100,000 population) [28], and the high adult HIV prevalence in Botswana [25], it is likely that our results point to NTM colonization or disease. It is worth noting that among NTM group just above 6% of patients were smear positive for AFB and this remain to be a concern in settings without sputum culture facility and smear is the only diagnostic test. Patients with smear positive for AFB and NTM positive culture are the likely to receive anti-TB treatment. The present study demonstrated high specificity (98.6%) of Xpert MTB/RIF among NTM positive samples. The concern of managing smear positive NTM patients with anti-TB treatment might be minimized if countries are shifting smear-based diagnostic algorithm to Xpert MTB-based diagnostic algorithm. To characterize the epidemiology of NTM and establish management strategy further study is essential in our settings.

In our study, the most common NTM species isolated was *M*. *intracellulare* followed b*y M*. *gordonae* and *M*. *malmoense*. Similar to reports from Hoza, Buijtels and Lan *et als*. [1, 7, 19], *M*. *intracellulare and M*. *gordonae* were among the top three species identified in culture-positive samples suggesting a similar predominance of these NTM species in different settings. In contrast to Hoza, Buijtels and Lan *et als*, where no *M*. *malmoense was reported*, *we* found *M*. *malmoense* (4%) among the common NTMs. Aliyu *et al in* Nigeria also reported *M*. *malmoense* (3%) among culture-positive mycobacteria identified with NTM isolates [6].

Identifying *M*. *malmoense* among patients with NTM disease has clinical significance, because of (1) this species may cause serious pulmonary morbidity reflecting a level of virulence unmatched by other NTM species [8], and (2) the two rpoB specific molecular beacons in *M*. *malmoense*, can give false positive Xpert MTB/RIF results with possible rifampicin resistance due to weak cross hybridization [13]. Rarely identified species of *M*. *lentiflavum* reported from Tanzania [1] and Zambia [7, 26] were also isolated in our setting. It is worth noting that *M*. *xenopi*, the third most frequent worldwide, was not identified in the present study and that was in agreement with Hoefsloot report which describes *M*. *Xenopi* as more common in Europe and Canada than in Southern Africa [8].

Studies from Sub-Saharan countries, including the present study, demonstrated that NTM species, *M*. *intracellulare*, *M*. *malmoense*, *M*. *abscessus and M*. *kansasi*, most commonly causing pulmonary infection are prevalent in Africa [1, 6, 7, 29]. Pulmonary NTM disease can have a similar presentation to pulmonary TB disease. The current TB diagnostic algorithms in Botswana do not include screening, diagnosis and management of NTM [14]. Therefore it remains difficult for clinicians to provide accurate diagnosis and treatment to patients with NTM disease. Hoza *et al* noted that not addressing NTM diagnostic concerns would lead to potential under treatment of NTM [1], and as Maiga *et al* reported, possible over-diagnosis and over-treatment of drug resistant TB as well, especially in cases where NTM infection or disease co-exists with drug sensitive TB [9].

In our settings more than one percent isolates (3/219) of culture-positive NTM Xpert MTB/RIF test results indicated MTB detected with rifampicin resistance. Though the high specificity (98.6%) of Xpert MTB/RIF test to rule out TB among NTMs was reassuring, the three test results with MTB detected and rifampicin resistance were concerning since it raises the possibility of multidrug-resistant TB diagnosis and treatment. The three patient with NTM species that detected and rifampicin resistant MTBC based on Xpert MTB/RIF included a patient with mixed NTM (*M*. *avium and intracellulare)* and two patients with mycobacterium species. The reason for the rifampicin resistance is unclear. For all the nine patients with *M*. *malmoense*, the species that has a potential for false Xpert MTB/RIF rifampicin resistance due to weak cross hybridization [13], MTB was not detected by Xpert MTB/RIF test.

Demographic characteristics of patients with NTM and MTBC were similar in this study, except age. Our finding is consistent with previous reports where age ≥50 was higher among patients with NTM than MTBC [6, 30, 31, 32]. Aliyu and Fusco-da-Costa *et al* reported that compared to patient with MTBC the average age among patients with NTM is higher [6, 30]. Prevots and Chung *et al* also described that generally the NTM prevalence increases with age compared to MTBC [31, 32], the likely reason being TB is a highly virulent pathogen, capable of infecting healthy individuals of all age and NTMs, in contrast, are opportunistic pathogens and patients need to have some risk factor for infection including age [30].

Clinically we observed that patients with MTBC positive culture were more symptomatic than NTM. In our study, fever, night sweats and weight loss were higher among patients with MTB. This is similar to reports from BaHammam *et al* (Riyadh, Saudi) and Kendall *et al* (Oregon (USA) [33, 34]. In the present study, however, the association became non-significant after adjusting for age, sex, CD4, BMI and history of TB in a multi variable logistic regression model (Table 1). Findings from our study were consistent with a previous report that anemia was more common among TB patients than NTM [35]. There is limited report so far that has described the association of anemia with NTM. The rare exception is the possibility of disseminated NTM infection that might occur among patients with sickle cell patients [36].

Our study has some limitations. First the results analysed were from symptomatic patients who were able to submit at least one sputum. Those patients who screened positive for TB symptom but who were not able to produce sputum were excluded and the prevalence of pulmonary NTM infection may be under reported. Second, the two assays (Hain GenoType CM and AS) used were not able to further speciate the type of NTM for 21% of the isolates. Third, the pulmonary specimens were collected from patients attending 22 HIV care and treatment clinics in 12 districts that do not necessarily represent the NTM prevalence in the whole country since the study facilities and patients included in this study were not randomly selected. However, these results do reflect a population of PLHIV enrolled in care and treatment that is typical in Botswana and findings provide useful epidemiological information about NTM infection that would be clinically relevant in the country. Fourth, both smoking and mine current or history of exposure as predisposing factors predisposing factors for NTM disease (or colonization); and our data was not complete to report the proportions and NTM and MTB. Last, though we were able to assess and rule out the potential contamination from water sources at our TB referral laboratory we were not able to rule out the NTM colonization or environmental contamination from peripheral clinics where sputa were collected.

In conclusion, in our settings, over-half (56%) of a positive sputum culture among PLHIV with TB symptoms was NTM. This study suggests sputum culture and species identification for TB symptom-positive PLHIV with negative Xpert MTB/RIF results remains important to facilitate possible NTM diagnosis and hasten time to appropriate treatment. Further research is needed to evaluate trends in NTM and MTB disease incidence in PLHIV, and to establish best management strategies for symptomatic Xpert-negative patients with presumptive TB.

## Supporting information

S1 Fig NTM isolation by month and year. (TIFF)
