# Supplementary figures and images for: Higher-than-expected prevalence of non-tuberculous mycobacteria in HIV setting in Botswana: Implications for diagnostic algorithms using Xpert MTB/RIF assay
Source: PLoS One. 2017 Dec 22;12(12):e0189981. doi: 10.1371/journal.pone.0189981 (PMC5741233; doi:10.1371/journal.pone.0189981)

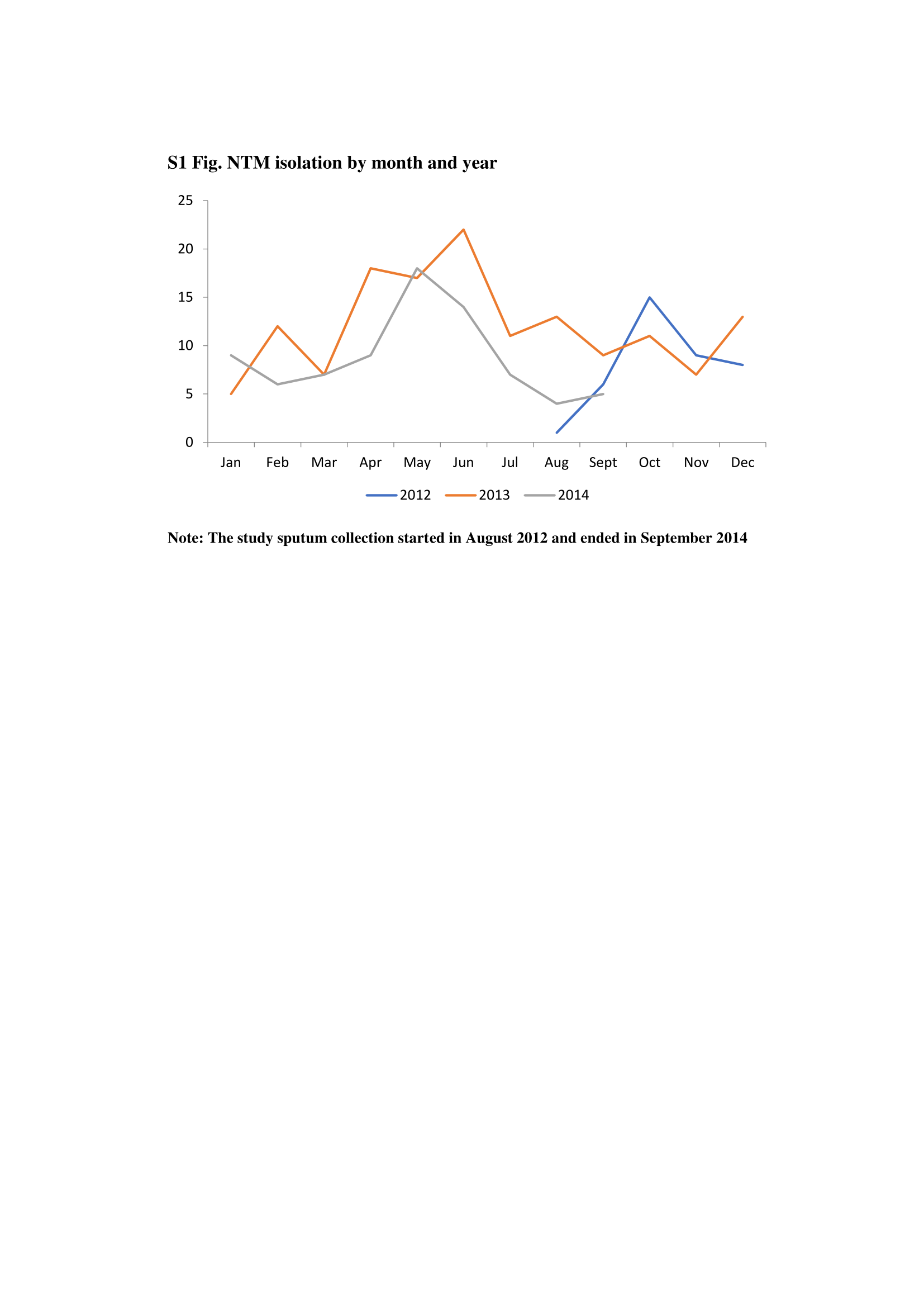

Supplement: S1 Fig — (TIFF) [file pone.0189981.s001.tiff]
